# Supplementary material for: Identification and Mapping of QTLs for Adult Plant Resistance in Wheat Line XK502
Source: Plants (Basel). 2024 Aug 25;13(17):2365. doi: 10.3390/plants13172365 (PMC11396990; doi:10.3390/plants13172365)
Supplement: Supplementary file 1 [file plants-13-02365-s001.zip › plants-3145151-supplementary.pdf]

Table S1. Quantitative trait locus of adult plant resistance to stripe rust detected in progenies of SY95-71/XK502 by using inclusive composite interval mapping in five environments.

Quantitative trait loci	Trait name	Marker interval	Genetic distance (cM)	Physical distance (Mb)	LOD[1]	PVE%[2]	Add[3]
QYrxk502.swust-1BL	22JY-IT	*AX-109335890—AX-109389405*	80.46-81.54	670.59-670.38	2.95	5.96	-0.54
	23JY-IT				10.35	19.64	-0.91
	24GY-IT				3.85	7.72	-0.67
	24JY-IT				6.05	11.89	-0.85
	22JY-DS				2.79	5.67	-7.33
	23JY-DS				10.30	19.60	-12.78
	24GY-DS				4.46	8.92	-10.74
	24JY-DS				5.11	10.17	-10.16
QYrxk502.swust-2BL	22JY-IT	*AX-108884194—AX-110024591*	361.45-362.20	690.21-683.29	4.83	10.04	-0.59
	23MY-IT				12.84	24.26	-1.01
	24GY-IT				21.44	36.74	-1.47
	24JY-IT				6.01	12.03	-0.86
	22JY-DS				4.74	9.63	-9.62
	23MY-DS				12.14	23.04	-13.69
	24GY-DS				19.91	34.50	-21.28
	24JY-DS				4.85	9.77	-10.03
QYrxk502.swust-3AS	22JY-IT	*AX-109274841—AX-109308178*	310.26-317.30	44.45-37.30	4.47	10.26	-0.74
	23JY-IT	*AX-111631905—AX-109308178*	307.78-317.30	46.01-37.30	4.68	10.88	-0.71
	22JY-DS	*AX-109274841—AX-109308178*	310.26-317.30	44.45-37.303	4.34	9.58	-9.97
	23JY-DS	*AX-111631905—AX-109308178*	307.78-317.30	46.01-37.30	4.95	11.30	-10.13
QYrxk502.swust-3BS	22JY-IT	*AX-108747357—AX-109438796*	47.20- 57.27	0.93- 7.53	9.24	19.82	-0.99
	23MY-IT				4.39	9.76	-0.64
	23JY-IT				7.56	17.81	-0.88
	24GY-IT				4.97	11.47	-0.82
	24JY-IT				9.03	20.79	-1.13
	22JY-DS				10.67	23.98	-15.19
	23MY-DS				5.01	11.27	-9.55
	23JY-DS				7.56	17.12	-12.07
	24GY-DS				5.80	14.66	-13.87
	24JY-DS				9.30	21.21	-14.78
QYrxk502.swust-7BS	22JY-IT	*AX-109968088—AX-110982135*	405.25-406.79	18.22-15.51	3.92	8.02	-0.63
	23MY-IT				6.09	11.90	-0.70
	23JY-IT				5.65	11.23	-0.69
	24GY-IT				4.70	9.31	-0.73
	24JY-IT				5.63	11.21	-0.82
	22JY-DS				4.31	8.81	-9.13
	23MY-DS				6.39	12.41	-9.96
	23JY-DS				4.67	9.33	-8.82
	24GY-DS				4.90	9.74	-11.22
	24JY-DS				5.76	11.49	-10.80

1 LOD, logarithm of odds score;

2 PVE, percentages of the phenotypic variance explained by individual QTL;

3 Add, additive effect of resistance allele.

Table S2. Excellent families screened for stripe rust and agronomic traits and the QTL carried by the families.

Genotype	Stripe rust		Agronomic trait							QTL				
	IT	DS	PH	PTN	SL	TKW	GL	GW	LWR	1BL	2BL	3AS	3BS	7BS
SY95-71	8.60	88.50	83.58	7.00	8.33	33.68	5.50	2.92	1.90	−[1]	−	−	−	−
XK502	2.00	0.90	97.75	9.00	10.38	44.12	6.62	3.14	2.12	+[2]	+	+	+	+
F_8-9	2.85	5.70	85.33	7.00	9.09	42.56	6.27	3.06	2.07	−	+	−	−	+
F_8-58	3.20	11.60	83.83	9.00	8.80	49.83	6.89	3.20	2.16	+	+	+	+	−
F_8-61	3.10	8.40	88.58	11.00	8.69	42.87	5.98	3.23	1.86	−	+	−	−	+
F_8-66	2.90	12.10	88.25	8.00	9.45	47.27	6.59	3.25	2.04	+	+	−	+	−
F_8-72	3.35	17.00	89.00	7.00	10.11	46.37	6.35	3.27	1.95	+	+	+	−	+
F_8-75	2.90	10.00	89.67	9.00	9.34	48.43	6.19	3.35	1.86	+	−	−	−	+
F_8-86	3.45	14.10	83.92	10.00	9.01	40.10	6.25	3.07	2.04	−	−	−	+	+
F_8-94	2.50	4.95	86.00	6.00	9.30	40.72	6.09	3.12	1.96	−	+	−	+	+
F_8-107	1.90	2.20	88.42	9.00	8.89	41.28	5.89	3.13	1.89	+	+	+	+	−
F_8-109	2.65	9.00	84.83	10.00	9.48	45.87	6.46	3.20	2.03	+	+	+	−	−
F_8-114	3.45	14.00	89.33	7.00	8.65	41.59	5.87	3.21	1.84	+	+	−	+	+
F_8-142	3.40	13.10	88.58	7.00	8.52	48.72	6.40	3.30	1.95	+	+	−	−	−
F_8-149	3.20	12.95	87.58	8.00	9.35	49.12	6.41	3.32	1.94	+	+	+	−	−
F_8-197	3.05	13.10	86.83	7.00	8.99	46.78	6.18	3.27	1.90	+	+	+	−	−
F_8-210	3.45	19.50	86.08	9.00	8.55	45.64	6.01	3.18	1.90	+	+	−	−	+
F_8-220	2.10	6.50	86.92	8.00	9.65	46.23	6.47	3.25	1.99	+	−	−	+	−

[1] − No quantitative trait loci (QTL) on corresponding chromosome

[2] + Quantitative trait loci (QTL) present on the corresponding chromosome.
